# Guideline adherence in bone-targeted treatment of cancer patients with bone metastases in Germany

**DOI:** 10.1007/s00520-019-05018-2

**Published:** 2019-08-14

**Authors:** Hartmut Link, Ingo Diel, Carsten-H. Ohlmann, Laura Holtmann, Markus Kerkmann, for the Associations Supportive Care in Oncology (AGSMO), Medical Oncology (AIO), Urological Oncology (AUO), within the German Cancer Society (DKG) and the German Osteooncological Society (DOG)

**Affiliations:** 1 Hämatologie, Onkologie, Pfaffplatz 10A, 67655 Kaiserslautern, Germany; 2 Praxisklinik am Rosengarten, Augustaanlage 7-11, 68165 Mannheim, Germany; 3 Malteser Krankenhaus Bonn/Rhein-Sieg, Von-Hompesch-Str.1, 53123 Bonn, Germany; 4 MMF GmbH, Heideblick 59, 44229 Dortmund, Germany

**Keywords:** Guideline adherence, Bone metastases, Osteolysis, Osteoprotection, RANK ligand inhibition, Denosumab, Bisphosphonate

## Abstract

**Purpose:**

To assess adherence to the current European Society for Medical Oncology (ESMO) clinical practice guideline on bone health in cancer patients and the German guidelines for lung, breast, and prostate cancer among German oncologists in hospitals and office-based physicians and to identify predictors of guideline compliance to assess the needs for dedicated training.

**Methods:**

This was a retrospective sample analysis representing hospitals and office-based physicians in Germany in 2016. Records from lung, breast, and prostate cancer patients who had received a diagnosis of bone metastasis between April 1, 2015, and March 31, 2016, were included. Oncologists at participating centers answered a self-assessment survey on aspects related to their professional life, including guideline adherence and years of clinical experience in medical oncology. Guideline adherence rates were assessed from patient records. Treatment variables and survey data were used to identify predictors of guideline compliance in a Classification and Regression Tree (CART) analysis.

**Results:**

Disregarding recommendations for supplementation of calcium and vitamin D, guideline adherence among physicians treating lung, breast, or prostate cancer patients was 62%, 92%, and 83%, respectively. Compliance was 15%, 42%, and 40% if recommendations for dietary supplements were taken into account. Identified predictors of guideline compliance included treatment setting, medical specialty, years of professional experience, and frequency of quality circle attendance.

**Conclusions:**

Compliance with the ESMO and the German guidelines in cancer patients varies between medical specialties. In particular, patients with lung cancer and bone metastases often do not receive the recommended osteoprotective treatment and required supplementation. Discrepancies between guideline recommendations and common practice should be addressed with dedicated training.

**Electronic supplementary material:**

The online version of this article (10.1007/s00520-019-05018-2) contains supplementary material, which is available to authorized users.

## Introduction

Bone metastases are common in advanced cancer and can be associated with clinically relevant morbidity, including fractures, pain, nerve compression, and hypercalcemia. The incidence of bone metastases is between 65 and 75% in patients with breast and prostate cancer and between 30 and 40% in patients with advanced lung cancer [1].

Skeletal-related events (SRE) are typical complications of the advanced situation and considerably impair the quality of life of the people affected. Fractures and the need for radiotherapy are the most frequently reported SREs. Far less known is the fact that patients with skeletal complications have a shorter survival time compared with patients with skeletal metastases without SRE [2, 3]. Moreover, patients with complications spend much more days in a hospital and cause significantly more treatment costs than those without skeletal events [4–6]. Not only for medical and ethical reasons but also for economic reasons, it is important to protect patients with bone metastases from skeletal complications. Although, the therapy of bone metastases needs an interdisciplinary approach, the basic therapy for patients with bone metastases is and remains the use of antiresorptives such as bisphosphonates and the RANKL inhibitor denosumab. Both substances have a direct effect on bone pain and significantly reduce skeletal events by improving bone stability.

Osteoprotective substances such as denosumab or bisphosphonates (BP) are recommended for drug treatment by the guidelines (GLs) most frequently used in Germany [7–9]. The ESMO-GL recommends to start bisphosphonates or denosumab as soon as bone metastases are definitively diagnosed in order to delay the first SRE and reduce subsequent complications from metastatic bone disease [8]. The GLs also recommend the supplementation of calcium and vitamin D in order to minimize the risk of hypocalcaemia through osteoprotective therapy. Similar recommendations are part of the German organ-specific S3 GLs. In addition, the German GLs recommend a dental check-up and treatment if necessary to prevent osteonecrosis of the jaw.

As an exception, in prostate cancer, the organ-specific German S3 GL recommends osteoprotective therapy depending on the stage of the disease, namely, whether the prostate carcinoma is still hormone-sensitive or whether it is a castration-resistant prostate cancer. Osteoprotective therapy is only recommended in the castration-resistant setting, as no clinical benefit for the patients could be proven in two studies at the hormone-sensitive stage. This recommendation is only valid for zoledronic acid. Data on the use of denosumab in hormone-sensitive prostate cancer are missing [9–11].

In addition, studies have shown that adjuvant osteoprotective substances such as clodronate also influence the tumor itself from the very beginning, both with regard to the occurrence of metastases and with regard to overall survival [12]. Even after a median follow-up of 97 months, clear advantages were still visible in overall survival [13].

This representative survey examined the implementation of the current ESMO guideline (2014) [8] on bone health for cancer patients with bone metastases (BM). In addition, the implementation of the osteoprotective recommendations from the respective national specialist S3 guidelines on lung, breast, and prostate cancer was examined and taken into account. In addition, the competence profile of the attending physicians was analyzed.

## Methods

The methods and analysis of this study have already been successfully applied and published in comparable studies of AGSMO (formerly ASORS) for neutropenia prophylaxis with G-CSF after chemotherapy [14, 19]. Further details are published in the supplementary material.

### Hypotheses

The following hypotheses were tested with the help of the retrospective, representative patient documentation and the physicians’ survey:

- As a core hypothesis, it was assumed that the guidelines for osteoprotection and therapy of osteolysis and osteoblastic metastases are insufficiently implemented in “everyday therapy.”
- The risk and consequences of osteolysis/osteoblastic metastases are not sufficiently known; i.e., the level of knowledge is not sufficient.
- The competence profile of the treating physicians correlates with the guideline-compliant osteoprotection, and the analysis of the competence profile of the physicians provides starting points for the education of further medical training in oncology.

### Representative sample (phase 1)

The representativity of the sample is guaranteed by the fact that it is based on a previously performed care structure analysis in the various oncological tumor entities, and the necessary sample size is determined on the basis of the respective extrapolated prevalence of patients with bone metastases in the individual tumor entities.

For a reliable sample representative of osteoprotective prophylaxis and therapy in patients with bone metastases in Germany, the target sample size was calculated to 1750 patients (breast carcinoma (BC), 800; lung carcinoma (LC), 400; prostate carcinoma (PC), 550), and the distribution of the patients to be documented among the participating institutions in the individual indications is determined. It is carried out on the basis of the collected data on patient numbers and the care structure data of the institution from phase I. Further details are given in the supplementary material.

### Patient documentation (phase 2)

In phase 2, the current course of therapy (surgery of BM, radiotherapy, endocrine therapies, chemotherapy, targeted therapies, and checkpoint inhibitors) of patients with bone metastases was recorded retrospectively and anonymously from the time of diagnosis of the metastases to the time of documentation based on the patient records. In addition, the bone-related complaints of the patients were recorded at two points in time, firstly 3 months after the diagnosis of BM and secondly at the time of documentation. The documentation started in September 2016, so the observation period varies between 6 and 18 months, due to the inclusion criteria. Bone-related complaints were rated on the following scale: pain-free or improvement of complaints, unchanged complaints, and worsening of complaints. Included were patients with osseous metastases in lung cancer (NSCLC/SCLC), breast cancer, or prostate cancer who were diagnosed with bone metastases for the first time between April 1, 2015, and March 31, 2016. Patients participating in studies on drug tumor therapy were allowed to be included in the survey.

Patients without confirmed osseous metastases and patients with hematological neoplasias were excluded.

To prevent selection bias in patient selection, all patients with bone metastases diagnosed until the assigned number of patients was reached were documented chronologically for each participating center from the set date.

### Survey of physicians (phase 3)

Parallel to patient documentation, in a third phase, the attending physicians of the centers participating in documentation (phase 2) were asked about their competence profile, their assessment of the guideline quality, and their practicability. The participation on this questionnaire was voluntary and the data of physicians was collected strictly pseudonymously.

### Definition of the standard and evaluation

The defined standard for guideline-adherent osteoprotection was based on the ESMO guideline [8]. Both a “weak” standard without consideration of concomitant medication and a “strict” standard were defined in which the concomitant medication recommended by the GL was taken into account. Two different degrees of deviations from the GL were defined (major/minor). GL-compliant therapy (“weak standard”) is defined as follows.

#### Treatment with bisphosphonates or denosumab

Treatment with bisphosphonates or denosumab is indicated for bone metastases. In prostate carcinoma, however, the German organ-specific S3 GL recommends osteoprotective therapy depending on the stage of the disease, namely, whether the prostate carcinoma is still hormone-sensitive or whether it is a castration-resistant prostate carcinoma. The recommendation that an osteoprotective therapy in the hormone-sensitive stage should be omitted was not yet implemented in the GL at the time of the survey, so that in the hormone-sensitive stage, both a therapy with and without bisphosphonates or denosumab are considered to comply with the GL. In the castration-resistant stage, GL-compliant therapy requires osteoprotective therapy with bisphosphonates or denosumab; its omission in this case constitutes a major GL deviation.

Denosumab is indicated/recommended for the treatment of all three diseases. The three indications also differ according to which bisphosphonates are considered GL-compliant (Table 1).

**Table 1:** Definition of the standard (“weak”)

	Lung cancer	Breast cancer	Prostate cancer
	Initiation of therapy with any of the following substances/latest start after diagnosis of bone metastases		
According to the guidelines	Zoledronate or denosumab/≤ 6 months	Zoledronate, clodronate, pamidronate, ibandronate, or denosumab/≤ 6 months	Zoledronate or denosumab/≤ 6 months
Minor deviation	Zoledronate or denosumab/> 6 months < 12 months or other (off label) bisphosphonates	Zoledronate, clodronate, pamidronate, ibandronate or denosumab/> 6 months < 12 months	Zoledronate or denosumab/> 6 months < 12 months^a^ or other (off label) bisphosphonates
Major deviation	Zoledronate or denosumab/> 12 months	Zoledronate, clodronate, pamidronate, ibandronate, or denosumab/> 12 months	Zoledronate or denosumab/> 12 months

^a^An exception is prostate cancer which is still hormone-sensitive at the diagnosis of bone metastases. The organ guideline (as of 2014) did not recommend osteoprotective therapy in this case

In lung cancer, breast cancer, and castration-resistant prostate cancer, treatment with bisphosphonate or denosumab is considered GL-compliant for up to 6 months after diagnosis of bone metastases. Within this time, a dental treatment to prevent osteonecrosis of the jaw should also be possible if necessary. An initiation more than 6 and less than 12 months after diagnosis of bone metastases represents a minor deviation from the GL; an initiation more than 12 months after diagnosis of bone metastases represents a major deviation. Prostate carcinoma is an exception (see above and Table 1).

Patients who died within 3 months after diagnosis of bone metastases are assumed to have had a pre-final stage, so that osteoprotective therapy no longer necessarily had to be initiated. Missing osteoprotective therapy in this group is considered GL-compliant.

### Statistical tests

The statistical data analysis was carried out using IBM SPSS Statistics 20.0 for Windows. The evaluation was primarily descriptive. For comparisons of interval-scaled variables, such as the clinical effect of osteoprotection, the Mann-Whitney *U* test was performed, if independent variables are binominal. In case of non-binominal independent variables, the Kruskal-Wallis test was used, supplemented by corresponding pairwise comparisons. In order to address the problem of inflation of type I errors (false-positive or α-errors) by multiple testing, the *p* values were adjusted using the Benjamini and Hochberg procedure to control the false discovery rate (FDR) [15]. Since the design of this study is explorative in character, correction of the FDR is more appropriate than a Bonferroni-based correction of the family-wise error rate (FWER). To determine the effect strength, the correlation coefficient was calculated using the following classification: *r* = 0.5 corresponds to a strong effect, *r* = 0.3 to a medium effect, and *r* = 0.1 to a low effect [16]. Frequency comparisons were made using the *χ*^2^ test.

In order to analyze the possible correlations between treatment in accordance with the guidelines and the competence profile of the treating physicians, the data of the patient documentation and the practitioner survey were correlated and analyzed using a Classification and Regression Tree (CART). CART is a tree-building binary recursive partitioning method that uses the Gini index for discrete distributions [17, 18]; for details, see earlier publication [14]. The following data from the physician survey are included in the evaluation: age of the treating physician; academic title; specialist medical training; training place and duration of training in oncology and in drug tumor therapy; position in the department/practice; activity in study groups; publications in specialist journals and textbooks; active cooperation in guidelines; scientific focus; participation in regional, national, and international congresses; and participation in training courses.

## Results

Target and actual sample size of the patients to be evaluated are breast carcinoma (BC, 800/803), prostate carcinoma (PC, 550/549), lung carcinoma (LC, 400/414), and total (1750/1766). One hundred twenty clinics and 130 practices with a total of 268 physicians participated.

### Clinical effect of osteoprotection (Table 2)

In the overall population, i.e., all observed indications, a positive correlation between osteoprotective therapy with bisphosphonates or denosumab and an improvement in bone-related complaints of patients can be observed after 3 months (*p* < 0.001) if osteoprotective therapy was started no later than 2 months after diagnosis of BM. However, the difference was only statistically significant in LC patients (*p* < 0.001, *r* = 0.291). A statistically significant difference in bone-related complaints before and after osteoprotective treatment was not observed for BC and PC patients (BC, *p* = 0.372; PC, *p* = 1). Differences in hormone sensitivity can be observed in PC, but these are not significant in hormone-sensitive PC (*p* = 1) or in castration-resistant PC (*p* = 0.813). The bone-related complaints were also recorded at the time of documentation (i.e., current therapy situation; at least 6 maximally 18 months after diagnosis of bone metastases). If osteoprotective therapy was continued until the time of documentation, this was associated with a significant improvement in symptoms, both in the overall population and in the individual indications (see Table 2). The effect strength is in the medium range for all indications.

**Table 2:** Clinical results of osteoprotective therapy at reporting date in patients with a least stable disease (2A) and in patients with progressive disease (2B)

	With osteoprotective therapy			Without osteoprotective therapy			Correlation coefficient	Unadjusted *p* value	Adjusted *p* value
	pts (%)			pts (%)					
	P+	P±	P−	P+	P±	P−	*r*	*p*	*p*
2A									
All indications	644 (72.5)	232 (26.1)	12 (1.4)	82 (59.0)	50 (36.0)	7 (5.0)	0.108	0.001	0.004
Lung cancer	67 (70.5)	27 (28.4)	1 (1.1)	13 (39.4)	16 (48.5)	4 (12.1)	0.305	< 0.001	< 0.001
Breast cancer	363 (71.9)	136 (26.9)	6 (1.2)	18 (58.1)	12 (38.7)	1 (3.2)	0.073	0.091	0.159
Prostate cancer	214 (74.3)	69 (24.0)	5 (1.7)	51 (68.0)	22 (29.3)	2 (2.7)	0.058	0.266	0.372
Hormone-sensitive prostate cancer	129 (80.6)	29 (18.1)	2 (1.3)	35 (76.1)	10 (21.7)	1 (2.2)	0.048	0.492	0.495
Castration-resistant prostate cancer	83 (69.7)	33 (27.7)	3 (2.5)	13 (59.1)	8 (36.4)	1 (4.5)	0.085	0.361	0.460
Prostate cancer with unknown hormone status	2 (22.2)	7 (77.8)	0 (0.0)	3 (42.9)	4 (57.1)	0 (0.0)	0.221	0.411	0.480
2B									
All indications	29 (19.7)	46 (31.3)	72 (49.0)	10 (7.1)	23 (16.4)	107 (76.4)	0.286	< 0.001	< 0.001
Lung cancer	4 (13.8)	8 (27.5)	17 (58.6)	8 (11.4)	10 (14.3)	52 (74.3)	0.141	0.163	0.254
Breast cancer	16 (21.9)	23 (31.5)	34 (46.6)	0 (0.0)	7 (25.0)	21 (75.0)	0.292	0.003	0.007
Prostate cancer	9 (20.0)	15 (33.3)	21 (46.7)	2 (4.8)	6 (14.3)	34 (81.0)	0.360	0.001	0.004
Hormone-sensitive prostate cancer	4 (23.5)	6 (35.3)	7 (41.2)	2 (8.0)	5 (20.0)	18 (72.0)	0.317	0.041	0.082
Castration-resistant prostate cancer	4 (16.0)	9 (36.0)	12 (48.0)	0 (0.0)	1 (6.7)	14 (93.3)	0.462	0.003	0.007
Prostate cancer with unknown hormone status	1 (33.3)	0 (0.0)	2 (66.7)	0 (0.0)	0 (0.0)	2 (100)	0.408	0.495	0.495

P+, painless/improvement of bone pain; P±, unchanged bone pain; P−, worsening of bone pain

All patients with evaluable data on bone pain. Patients who are lost to follow-up or deceased within 6 months after diagnosis of bone metastases were excluded

### Guideline adherence

**Fig. 1:**
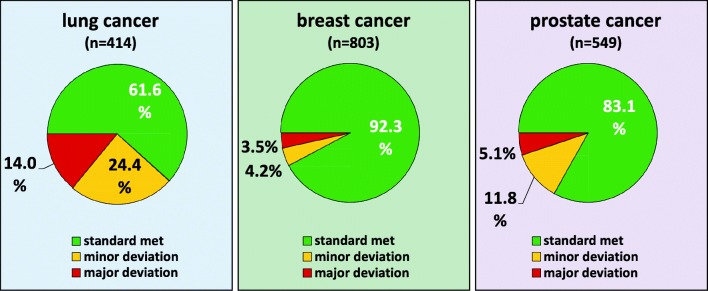
Guideline adherence in bone-targeted therapy (“weak standard”)

**Fig. 2:**
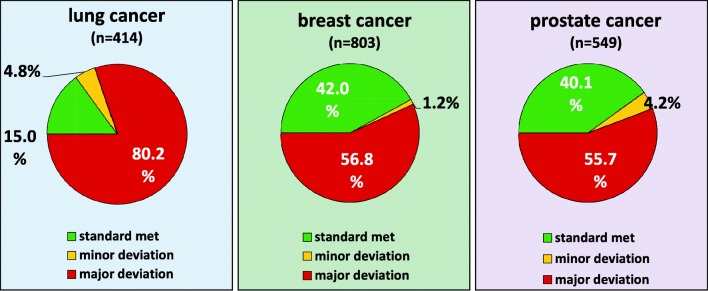
Guideline adherence in bone-targeted therapy (“strict standard”)

Bone pain improvement was correlated with guideline adherence (“weak standard”) in lung cancer (*p* = 0.012) and breast cancer (*p* = 0.007) but not in prostate cancer (*p* = 0.758) (see Table 3). The pairwise comparisons in the indications LC and BC show different results with respect to major and minor deviations, which are however not significant at the adjusted α level but in part show a trend. While in LC differences are to be found between standard met and major deviation (*r* = 0.212, *p* = 0.060) and between major and minor deviation (*r* = 0.275, *p* = 0.055), there are no differences between standard met and minor deviations (*r* = 0.039, *p* = 1). In contrast, no difference can be found between major and minor deviations in BC (*r* = 0.034, *p* = 1), whereas slight differences with weak effect size are found between standard met and major (*r* = 0.091, *p* = 0.182) as well as minor deviation (*r* = 0.091, *p* = 0.168). A possible explanation for this could be that a large proportion of minor deviation in the LC indication is due to the use of a bisphosphonate not recommended or approved by the GL in this indication, which may nevertheless have similar efficacy. In BC, the number of patients not treated in compliance with GL (“weak standard”) is small (major dev. *n* = 34, minor dev. *n* = 28), so that the differences may be underestimated. There are clear differences in the indications investigated (see Figs. 1 and 2). The bisphosphonates being used outside of their labeled indications are presented in Figure 3 in the supplementary material.

**Table 3:** Guideline adherence (“weak standard”) and bone pain at reporting date

3	Standard met			Minor deviation			Major deviation			Correlation coefficient	Unadjusted p value	Adjusted *p* value
	pts (%)			pts (%)			pts (%)					
	P+	P±	P−	P+	P±	P−	P+	P±	P−	*r*	*p*	*p*
All indications	699 (60.2)	307 (26.4)	156 (13.4)	76 (52.1)	40 (27.4)	30 (20.5)	24 (34.8)	22 (31.9)	23 (33.3)	0.121	< 0.001	< 0.001
Lung cancer	54 (40.6)	40 (30.1)	39 (29.3)	32 (47.1)	16 (23.5)	20 (29.4)	7 (21.9)	7 (21.9)	18 (56.3)	0.103	0.116	0.180
Breast cancer	397 (63.6)	169 (27.1)	58 (9.3)	11 (42.3)	10 (38.5)	5 (19.2)	5 (33.3)	8 (53.3)	2 (13.3)	0.122	0.002	0.009
Prostate cancer	248 (61.2)	98 (24.2)	59 (14.6)	33 (63.5)	14 (26.9)	5 (9.6)	12 (54.5)	7 (31.8)	3 (13.6)	− 0.005	0.907	0.907
Hormone-sensitive prostate cancer	149 (67.1)	49 (20.7)	29 (12.2)	21 (80.8)	5 (19.2)	0 (0.0)	0 (0.0)	0 (0.0)	0 (0.0)	− 0.101	0.101	0.177
Castration-resistant prostate cancer	86 (56.6)	40 (26.3)	26 (17.1)	10 (43.5)	8 (34.8)	5 (21.7)	10 (58.8)	5 (29.4)	2 (11.8)	0.035	0.628	0.733
Prostate cancer with unknown hormone status	3 (18.8)	9 (56.3)	4 (25.0)	2 (66.7)	1 (33.3)	0 (0.0)	2 (40.0)	2 (40.0)	1 (20.0)	− 0.257	0.225	0.315

P+, painless/improvement of bone pain; P±, unchanged bone pain; P−, worsening of bone pain

All patients with evaluable data on bone pain. Patients who are lost to follow-up or deceased within 6 months after diagnosis of bone metastases were excluded

Taking into account the accompanying medication (“strict standard”), the improvement of bone-related pain in all indications is associated with a GL-compliant therapy (Table 4). The Kruskal-Wallis test shows significant differences in all three indications: all indications (*p* < 0.001), LC (*p* = 0.006), BC (p = 0.006), and PC (*p* = 0.043). The pairwise comparisons show significant differences between standard met and major deviation for the entire spectrum (*r* = 0.137, *p* < 0.001) and BC (*r* = 0.109, *p* = 0.05) at low effect strength and differences between major and minor deviation for LC and mean effect strength (*r* = 0.230, *p* = 0.044).

**Table 4:** Guideline adherence (“strict standard”) and bone pain at reporting date

4	Standard met			Minor deviation			Major deviation			Correlation coefficient	Unadjusted *p* value	Adjusted *p* value
	pts (%)			pts (%)			pts (%)					
	P+	P±	P−	P+	P±	P−	P+	P±	P−	*r*	*p*	*p*
All indications	351 (65.9)	121 (22.7)	61 (11.4)	31 (68.9)	9 (20.0)	5 (11.1)	417 (52.2)	239 (29.9)	143 (17.9)	0.137	< 0.001	< 0.001
Lung cancer	19 (45.2)	13 (31.0)	10 (23.8)	13 (81.3)	1 (6.3)	2 (12.5)	61 (34.9)	49 (28.0)	65 (37.1)	0.170	0.009	0.025
Breast cancer	200 (68.7)	67 (23.0)	24 (8.2)	2 (22.2)	6 (66.7)	1 (11.1)	211 (57.8)	114 (31.2)	40 (11.0)	0.106	0.006	0.021
Prostate cancer	132 (66.0)	41 (20.5)	27 (13.5)	16 (80.0)	2 (10.0)	2 (10.0)	145 (56.0)	76 (29.3)	38 (14.7)	0.092	0.045	0.090
Hormone-sensitive prostate cancer	73 (68.9)	21 (19.8)	12 (11.3)	10 (100.0)	0 (0.0)	0 (0.0)	97 (66.0)	33 (22.4)	17 (11.6)	0.035	0.570	0.726
Castration-resistant prostate cancer	58 (63.7)	19 (20.9)	14 (15.4)	6 (60.0)	2 (20.0)	2 (20.0)	42 (46.2)	32 (35.2)	17 (18.7)	0.151	0.037	0.086
Prostate cancer with unknown hormone status	1 (33.3)	1 (33.3)	1 (33.3)	0 (0.0)	0 (0.0)	0 (0.0)	6 (28.6)	11 (52.4)	4 (19.9)	− 0.040	0.854	0.907

P+, painless/improvement of bone pain; P±, unchanged bone pain; P−, worsening of bone pain

All patients with evaluable data on bone pain. Patients who are lost to follow-up or deceased within 6 months after diagnosis of bone metastases were excluded

Overall, in patients with improvement of bone pain, the percentage of patients with osteoprotective therapy was significantly higher, with (54%) and without (47%) additional systemic or radiotherapy than in patients with unchanged or worsening pain (see Figure 4A (supplementary material)). Similar effects are found in the three cancers analyzed (supplement Figure 4B–D).

### Lung cancer

Without taking the accompanying medication (calcium and vitamin D) into account, 61.6% of patients with lung cancer received osteoprotective treatment in accordance with the guidelines. Minor deviations were observed in 24.4% of patients; major deviations from GL recommendations were seen in 14.0% of patients. There is a significant difference between certified centers (OnkoZert, DGHO, or CCC; 69.1%) and non-certified centers (56.1%) (*p* < 0.001). However, certified centers recorded more major deviations (19.4% vs 10.0%) but significantly fewer minor deviations (11.4% vs 33.9%) than non-certified centers.

### Breast cancer

92.3% of BC patients were treated according to GL; in 4.2% of patients, there were minor deviations; and in 3.5% of patients, there were major deviations from the GL. A statistically significant difference between certified (92.1%) and non-certified centers (92.5%) was not observed (*p* = 0.086).

### Prostate cancer

83.1% of PC patients were treated according to GL; in 11.8% of patients, there were minor deviations; and in 5.1% of patients, there were major deviations from GL-recommended treatment. There was no statistically significant difference between certified (80.2%) and non-certified centers (83.7%) (*p* = 0.285).

### Substitution of calcium and vitamin D (strict standard)

If calcium and vitamin D are taken into account as concomitant medication (as major deviation), 15.0% of patients with lung cancer were treated according to GL, 4.8% had minor deviations, and 80.2% had major deviations.

42.0% of BC patients were treated in accordance with guidelines, 1.2% had minor deviations, and 56.8% had major deviations.

40.1% of PC patients were treated according to GL; in 4.2% of patients, there were minor deviations, and in 55.7% of patients, there were major deviations, (see Fig. 2).

### Guideline adherence of participants (weak standard)

In patients treated in certified or comprehensive cancer centers (CERT), the guideline adherence was 85% vs 80% in other centers (*p* = 0.025). Guideline adherence differed between organ-specific oncologists and hematologist-oncologists (86% vs 76%, *p* < 0.001) and between hospital- and office-based physicians (78% vs 86%, *p* < 0.001).

### Guideline adherence of participants (strict standard)

Taking the supplements of vitamin D and calcium into consideration, guideline adherence was 39% in CERT vs 32% in other centers (*p* = 0.013). Guideline adherence in organ-specific oncologists and hematologist-oncologists was 42% vs 22% (*p* < 0.001) and 34% vs 36% in hospital- and office-based physicians, respectively (*p* = 0.3).

### Physicians

When asked to assess their own GL adherence, 70.1% of physicians stated they adhered completely, and 24.6% said they adhered partially to GL.

Classification and Regression Tree (CART) analysis split treatment by gynecologists and urologists from general oncologists or lung cancer specialists (GOSL): guideline adherence was 48% vs 21.8%, *p* < 0.001. Gyneco- or urological oncologists were split attending ≤ 3 or more national congresses (guideline adherence 52.8% vs 24.3%, *p* < 0.001); guideline adherence in GOSL experienced in medical tumor therapy for ≤ 15 years or more was 43.4% vs 15.3%, *p* < 0.001. The latter group was split by attendance to no or ≥ 1 quality circles per year, 29.5% vs 9.4%, *p* = 0.001.

## Discussion

Guideline adherence differs between the cancers examined. Both in pure osteoprotective therapy and taking into account recommended calcium and vitamin D supplements, the results in lung cancer are worse than in breast cancer and prostate cancer. This is in line with the results of studies on the adherence to G-CSF prophylaxis GL [14, 19]. In patients with breast or prostate carcinoma, the adherence to guidelines for osteoprotection is significantly higher, even if the absolute figures can still be significantly improved when analyzed according to strict standards.

The reasons for these differences are not clear. It probably plays a role that the prognosis of patients with lung cancer was worse compared with the two other carcinomas just a few years ago, and therefore, less importance is attached to osteoprotection.

Even if the osteoprotective therapy in metastatic breast and prostate carcinoma is 95% and above according to the so-called weak criteria, the question arises why 3–5% of those affected did not receive antiresorptive treatment.

Possibly the concern of side effects plays a role. Uncertainty was so great in the previous years that many oncologists were skeptical about antiresorptive treatment and sometimes stopped it.

Other reasons could be ignorance of guidelines or personal judgment (“Patient is symptom-free, patient is in a very advanced stage of the disease, with permanent bedriddenness, therapy too expensive, etc.”).

As far as compliance with the strict criterion is concerned, the situation is more difficult. Although GLs and product information recommend the addition of calcium and vitamin D to antiresorptives, the need to avoid a possible hypocalcaemia is ignored by many physicians.

This may be because physicians are not aware of the GLs and product information. The situation is slightly different for the administration of vitamin D. Vitamin D is necessary for numerous metabolic processes, especially for the reabsorption of calcium from the intestines. In this respect, cholecalciferol should be supplemented even if there is an adequate supply of calcium. This applies in particular to tumor patients [20].

Certified cancer centers showed significantly better adherence to GL for the weak standard only in lung cancer. If all diagnoses were analyzed together according to the strict standard, the overall results for certified centers were significantly better than not certified centers, while organ-specific centers were significantly better than hematological-oncological centers.

A study published after the end of our analysis shows that treatment with zoledronate every 12 weeks instead of 4 weeks did not lead to a higher risk of SRE [21]. In our trial, more than 90% of patients received the drugs every 3–4 weeks, as recommended so far.

It is interesting to note that 70.1% of the participating doctors said they adhered completely and 24.6% in part to the GL. This is contradicted by the present study results. This means that there is a discrepancy between the self-perception of physicians and their professional routine in supportive therapy.

The CART analysis shows that there is a high need for training among hematologist-oncologists and lung cancer specialists. Frequent participation in congresses is inversely correlated with GL adherence in gyneco- and urological oncologists. This could mean that supportive therapy does not play a trend-setting role at congresses. The frequent congress visitors must therefore be trained as a target group for osteoprotective therapy. It is incomprehensible that the longer professional experience and more frequent participation in quality circles goes hand in hand with poorer adherence to GL. Obviously, this describes a group of physicians who have a certain distance to osteoprotective supportive therapy. It can be seen that the CART analysis defines groups of doctors for whom training is particularly urgent.

It is one of the limitations of the study that no patient-reported outcome or bone symptoms like patient’s diary are feasible because of the study design. Since the ESMO guideline was published in 2014, the retrospective observation started in early 2015 to enable a broader GL awareness. In consequence, the observation time is limited on the one hand and varies on the other. The variation is caused by the inclusion period, which was chosen broadly to allow all sizes of facilities treating patients with bone metastases to generate representative real-world data.

It should be clear to all oncologists that osteoprotective therapy and related supplementation of calcium and vitamin D is indispensable in patients with bone metastases to avoid skeletal-related complications and to postpone their occurrence during the course of the disease [22]. As a general conclusion, guidelines must be combined with an effective concept and strategy of implementation.

## Electronic supplementary material

ESM 1 (PDF 118 kb)

ESM 2 (PDF 42 kb)

ESM 3 (PDF 345 kb)

## References

[1] Coleman RE (1997). Skeletal complications of malignancy. Cancer..

[2] Yong M, Jensen AO, Jacobsen JB, Norgaard M, Fryzek JP, Sorensen HT (2011). Survival in breast cancer patients with bone metastases and skeletal-related events: a population-based cohort study in Denmark (1999-2007). Breast Cancer Res Treat.

[3] Norgaard M, Jensen AO, Jacobsen JB, Cetin K, Fryzek JP, Sorensen HT (2010). Skeletal related events, bone metastasis and survival of prostate cancer: a population based cohort study in Denmark (1999 to 2007). J Urol.

[4] Barlev A, Song X, Ivanov B, Setty V, Chung K (2010). Payer costs for inpatient treatment of pathologic fracture, surgery to bone, and spinal cord compression among patients with multiple myeloma or bone metastasis secondary to prostate or breast cancer. J Manag Care Pharm.

[5] Yong C, Onukwugha E, Mullins CD (2014). Clinical and economic burden of bone metastasis and skeletal-related events in prostate cancer. Curr Opin Oncol.

[6] Pereira J, Body JJ, Gunther O, Sleeboom H, Hechmati G, Maniadakis N, Terpos E, Acklin YP, Finek J, von Moos R (2016). Cost of skeletal complications from bone metastases in six European countries. J Med Econ.

[7] Supportive Therapie bei onkologischen PatientInnen - Langversion 1.0, 2016, AWMF Registernummer: 032/054OL2016 18.4.2019 30.12.2017]. Available from: https://www.leitlinienprogramm-onkologie.de/leitlinien/supportive-therapie/. Accessed 4 Dec 2018

[8] Coleman R, Body JJ, Aapro M, Hadji P, Herrstedt J, Group EGW (2014). Bone health in cancer patients: ESMO clinical practice guidelines. Ann Oncol.

[9] Interdisziplinäre Leitlinie der Qualität S3 zur Früherkennung, diagnose und Therapie der verschiedenen Stadien des Prostatakarzinom. Berlin 2018 4/2019]. Available from: http://www.leitlinienprogramm-onkologie.de/leitlinien/prostatakarzinom/. Accessed 4 Dec 2018

[10] Miriam H, Jens B, Arnulf S, Tilman T (2017). Denosumab treatment in the management of patients with advanced prostate cancer: clinical evidence and experience. Ther Adv Urol.

[11] Alibhai SMH, Zukotynski K, Walker-Dilks C, Emmenegger U, Finelli A, Morgan SC, Hotte SJ, Tomlinson GA, Winquist E (2017). Bone health and bone-targeted therapies for nonmetastatic prostate cancer: a systematic review and meta-analysis. Ann Intern Med.

[12] Diel IJ, Solomayer EF, Costa SD, Gollan C, Goerner R, Wallwiener D, Kaufmann M, Bastert G (1998). Reduction in new metastases in breast cancer with adjuvant clodronate treatment. N Engl J Med.

[13] Diel IJ, Jaschke A, Solomayer EF, Gollan C, Bastert G, Sohn C, Schuetz F (2008). Adjuvant oral clodronate improves the overall survival of primary breast cancer patients with micrometastases to the bone marrow: a long-term follow-up. Ann Oncol.

[14] Link H, Nietsch J, Kerkmann M, Ortner P (2016). Adherence to granulocyte-colony stimulating factor (G-CSF) guidelines to reduce the incidence of febrile neutropenia after chemotherapy - a representative sample survey in Germany. Support Care Cancer.

[15] Benjamini Y, Hochberg Y (1995). Controlling the false discovery rate: a practical and powerful approach to multiple testing. J R Stat Soc Ser B Methodol.

[16] Cohen J (1988). Statistical power analysis for the behavioral sciences.

[17] Breiman L, Friedman JH, Olshen RA, Stone CJ (1984). Classification and regression trees.

[18] Breiman L (2001). Statistical modeling: the two cultures (with comments and a rejoinder by the author). Stat Sci.

[19] Link Hartmut, Kerkmann Markus, Holtmann Laura, Ortner Petra (2018). G-CSF guideline adherence in Germany, an update with a retrospective and representative sample survey. Supportive Care in Cancer.

[20] Hadji P (2015). Cancer treatment-induced bone loss in women with breast cancer. BoneKEy Rep.

[21] Himelstein AL, Foster JC, Khatcheressian JL, Roberts JD, Seisler DK, Novotny PJ, Qin R, Go RS, Grubbs SS, O’Connor T, Velasco MR, Weckstein D, O’Mara A, Loprinzi CL, Shapiro CL (2017). Effect of longer-interval vs standard dosing of zoledronic acid on skeletal events in patients with bone metastases: a randomized clinical trial. JAMA..

[22] Menshawy A, Mattar O, Abdulkarim A, Kasem S, Nasreldin N, Menshawy E, Mohammed S, Abdel-Maboud M, Gadelkarim M, el Ashal GG, Elgebaly AS (2018). Denosumab versus bisphosphonates in patients with advanced cancers-related bone metastasis: systematic review and meta-analysis of randomized controlled trials. Support Care Cancer.

