# Supplementary material for: Guideline adherence in bone-targeted treatment of cancer patients with bone metastases in Germany
Source: Support Care Cancer. 2019 Aug 14;28(5):2175–84. doi: 10.1007/s00520-019-05018-2 (PMC7083811; doi:10.1007/s00520-019-05018-2)
Supplement: Supplementary file 1 — (PDF 118 kb) [file 520_2019_5018_MOESM1_ESM.pdf]

Supplementary material methods

Guideline adherence in bone targeted treatment of cancer patients with bone metastases in Germany

Hartmut Link[1]

Ingo Diel[2]

Carsten-H. Ohlmann[3]

Laura Holtmann[4]

Markus Kerkmann[4]

for the Associations Supportive Care in Oncology (AGSMO), Medical Oncology (AIO), Urological Oncology (AUO), within the German Cancer Society (DKG) and the German Osteooncological Society (DOG)

[1] Hämatologie, Onkologie, Pfaffplatz 10A, D- 67655 Kaiserslautern

+49 631 14102, hlink@kabelmail.de

ORCID 0000-0002-9659-0265

[2] Praxisklinik am Rosengarten, Augustaanlage 7-11, D-68165 Mannheim

[3]Malteser Krankenhaus Bonn/Rhein-Sieg, Von-Hompesch-Str.1, 53123 Bonn

[4]MMF GmbH, Heideblick 59, D-44229 Dortmund

all in Germany

Supplementary material methods

Methods

Representative sample (Phase 1)

In a first step data is collected to analyze the health care structure regarding the treatment of patients with bone metastases in Germany. In phase 1 all centers that potentially treat patients with lung, breast or prostate cancer are contacted and data of its facility care level and its number of treated patients is recorded using a one-sided pen-to-paper form. In addition, the willingness of care facilities to become involved in patient documentation is elicited (phase 2). To achieve a reliable, representative sample of patients treated in Germany, the sample size is calculated based on projection of the prevalence of patients with bone metastases in the individual indications (Table S1 supplementary material). The projection is based on the data issued by the Robert Koch Institute on deaths in the individual indications (23), the share of patients with osseous metastases and the median survival time following the diagnosis of bone metastases (3, 24-27). The target sample is guided by the maximum values and corresponds to approx. 3% of the projected prevalence with bone metastases.

The distribution of cases to be documented is specified in the individual indications amongst the facilities involved.

The participating centers are assigned to clusters on the basis of important differentiating features (facility type, care level, specialty and number of patients treated). The number of patients to be documented (phase 2) in the respective indication is assigned so that their share in the sample corresponds representatively to the surveyed care structure. Phase 1 involved 747 institutions (408 clinics and 339 practices) that reported data on their patient numbers. The physicians or the study staff filled in the questionnaire, they did not know the type of the analyses planned.

23. Krebs in Deutschland für 2013/2014. 2017; Berlin: Robert Koch-Institut; 2017. https://doi.org/10.17886/rkipubl-2017-007

24. Mediane Überlebenszeit nach Diagnose der Knochenmetastasen. München: Tumorregister München; 2013.
https://www.tumorregister-muenchen.de/facts/spec/spec_C50f__15_20170330_litvergl.pdf
https://www.tumorregister-muenchen.de/facts/spec/spec_C61__07_20131217_progr.pdf
https://www.tumorregister-muenchen.de/facts/surv/sC3334G-ICD-10-C33-C34-Lungentumor-Survival.pdf

25. Solomayer EF, Diel IJ, Meyberg GC, Gollan C, Bastert G. Metastatic breast cancer: clinical course, prognosis and therapy related to the first site of metastasis. Breast Cancer Res Treat. 2000;59(3):271-8.

26. Cetin K, Christiansen CF, Jacobsen JB, Nørgaard M, Sørensen HT. Bone metastasis, skeletal-related events, and mortality in lung cancer patients: A Danish population-based cohort study. Lung Cancer. 2014;86(2):247-54.

27. Jensen AO, Jacobsen JB, Norgaard M, Yong M, Fryzek JP, Sorensen HT. Incidence of bone metastases and skeletal-related events in breast cancer patients: a population-based cohort study in Denmark. BMC Cancer. 2011;11:29.
