# Supplementary material for: Guideline adherence in bone-targeted treatment of cancer patients with bone metastases in Germany
Source: Support Care Cancer. 2019 Aug 14;28(5):2175–84. doi: 10.1007/s00520-019-05018-2 (PMC7083811; doi:10.1007/s00520-019-05018-2)
Supplement: Supplementary file 2 — (PDF 42 kb) [file 520_2019_5018_MOESM2_ESM.pdf]

Supplementary material Tables S1-S3

Guideline adherence in bone targeted treatment of cancer patients with bone metastases in Germany

Hartmut Link[1]

Ingo Diel[2]

Carsten-H. Ohlmann[3]

Laura Holtmann[4]

Markus Kerkmann[4]

for the Associations Supportive Care in Oncology (AGSMO), Medical Oncology (AIO), Urological Oncology (AUO), within the German Cancer Society (DKG) and the German Osteooncological Society (DOG)

[1] Hämatologie, Onkologie, Pfaffplatz 10A, D- 67655 Kaiserslautern

+49 631 14102, hlink@kabelmail.de

ORCID 0000-0002-9659-0265

[2] Praxisklinik am Rosengarten, Augustaanlage 7-11, D-68165 Mannheim

[3]Malteser Krankenhaus Bonn/Rhein-Sieg, Von-Hompesch-Str.1, 53123 Bonn

[4]MMF GmbH, Heideblick 59, D-44229 Dortmund

all in Germany

Supplementary material Tables S1-S3

Table S1: Target sample size calculation

tumor localization	deaths 2010	patient share bone metastases		survival time (median)	prevalence with bone metastases		target sample size
	pts	min	max	months	min	max	pts
breast cancer	17573	70%	85%	1.83	22552	27385	~ 800
prostate cancer	12676	60%	80%	1.75	13310	17746	~ 550
lung cancer	43008	40%	60%	0.5	8602	12902	~ 400
TOTAL	73257	40%	85%	0.5-1.83	44068	58033	~ 1750

Table S2: Responder to phase I (health care structure): Number of centers and patients with bone metastases in 2nd half-year 2015

	Hospitals		office-based physicians		Total	
	centers	pts	centers	pts	centers	Pts
pulmonology	85	749	5	33	90	782
gynecology	158	7016	61	931	219	7947
urology	88	1193	192	2730	280	3923
general hematology / oncology	77	925	81	1777	158	2702
TOTAL	408	9883	339	5471	747	15354

Table S3: Federal state of participating centers and patients in phase 2 (patient documentation)

Federal state of Germany	centers	pts
Berlin	17	138
Brandenburg	7	36
Bremen	1	4
Hamburg	5	24
Mecklenburg-Vorpommern	6	31
Niedersachsen	27	288
Schleswig-Holstein	5	24
Hessen	16	102
Nordrhein-Westfalen	53	410
Sachsen	23	119
Sachsen-Anhalt	11	63
Thüringen	6	23
Baden-Württemberg	26	160
Bayern	38	252
Rheinland-Pfalz	4	47
Saarland	5	45
TOTAL	250	1766
