# Supplementary material for: Guideline adherence in bone-targeted treatment of cancer patients with bone metastases in Germany
Source: Support Care Cancer. 2019 Aug 14;28(5):2175–84. doi: 10.1007/s00520-019-05018-2 (PMC7083811; doi:10.1007/s00520-019-05018-2)

Guideline adherence in bone targeted treatment of cancer patients with bone metastases in Germany

Hartmut Link[1]

Ingo Diel[2]

Carsten-H. Ohlmann[3]

Laura Holtmann[4]

Markus Kerkmann[4]

for the Associations Supportive Care in Oncology (AGSMO), Medical Oncology (AIO), Urological Oncology (AUO), within the German Cancer Society (DKG) and the German Osteooncological Society (DOG)

[1] Hämatologie, Onkologie, Pfaffplatz 10A, D- 67655 Kaiserslautern
+49 631 14102, hlink@kabelmail.de

[2] Praxisklinik am Rosengarten, Augustaanlage 7-11, D-68165 Mannheim

[3]Malteser Krankenhaus Bonn/Rhein-Sieg, Von-Hompesch-Str.1, 53123 Bonn

[4]MMF GmbH, Heideblick 59, D-44229 Dortmund

all in Germany

Figure 3: Use of different bisphosphonates in lung, breast and prostate cancer. Pts with osteoprotective treatment

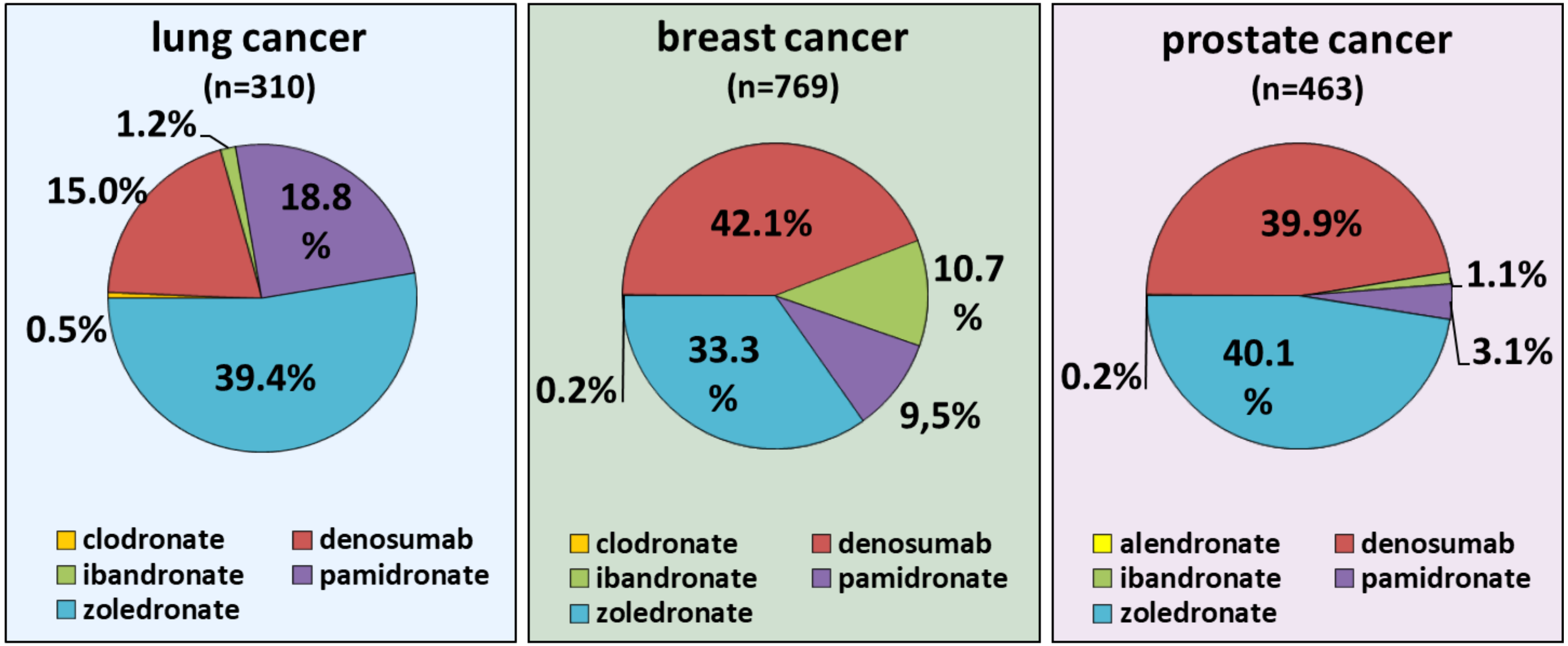

Figure 4A: systemic or radiotherapy, osteoprotective treatment and bone pain

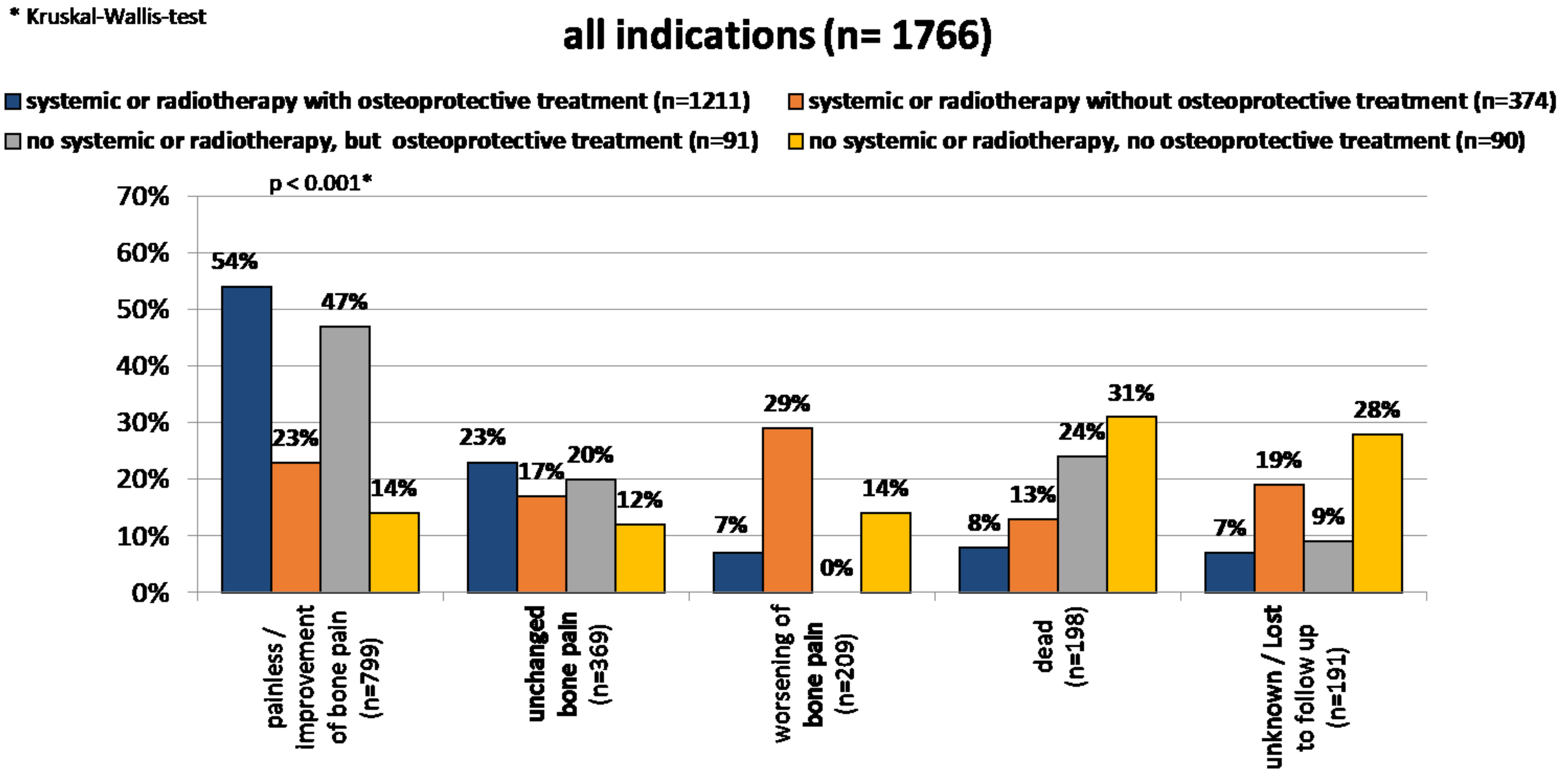

Figure 4B: systemic or radiotherapy, osteoprotective treatment and bone pain

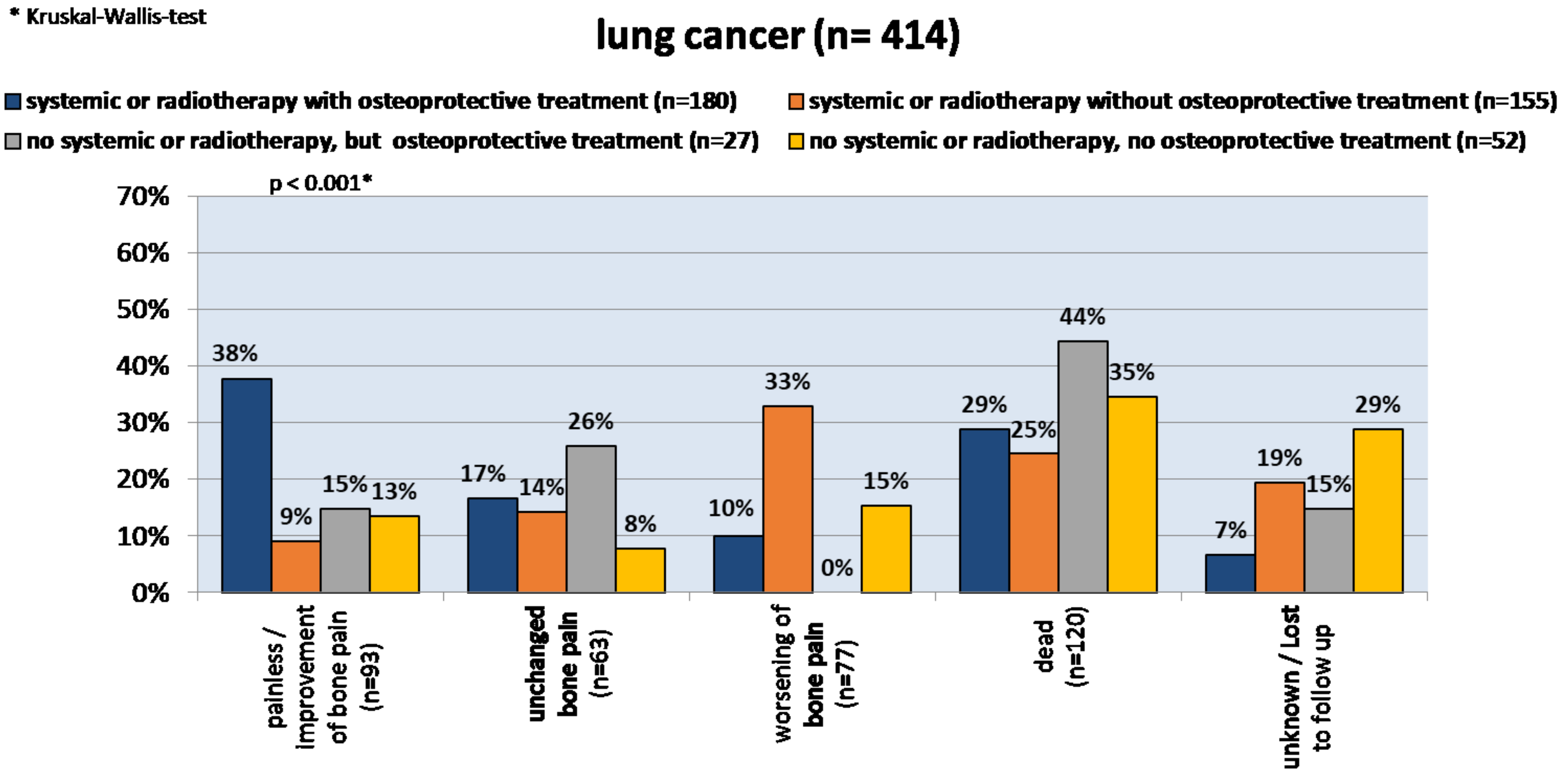

Figure 4C: systemic or radiotherapy, osteoprotective treatment and bone pain

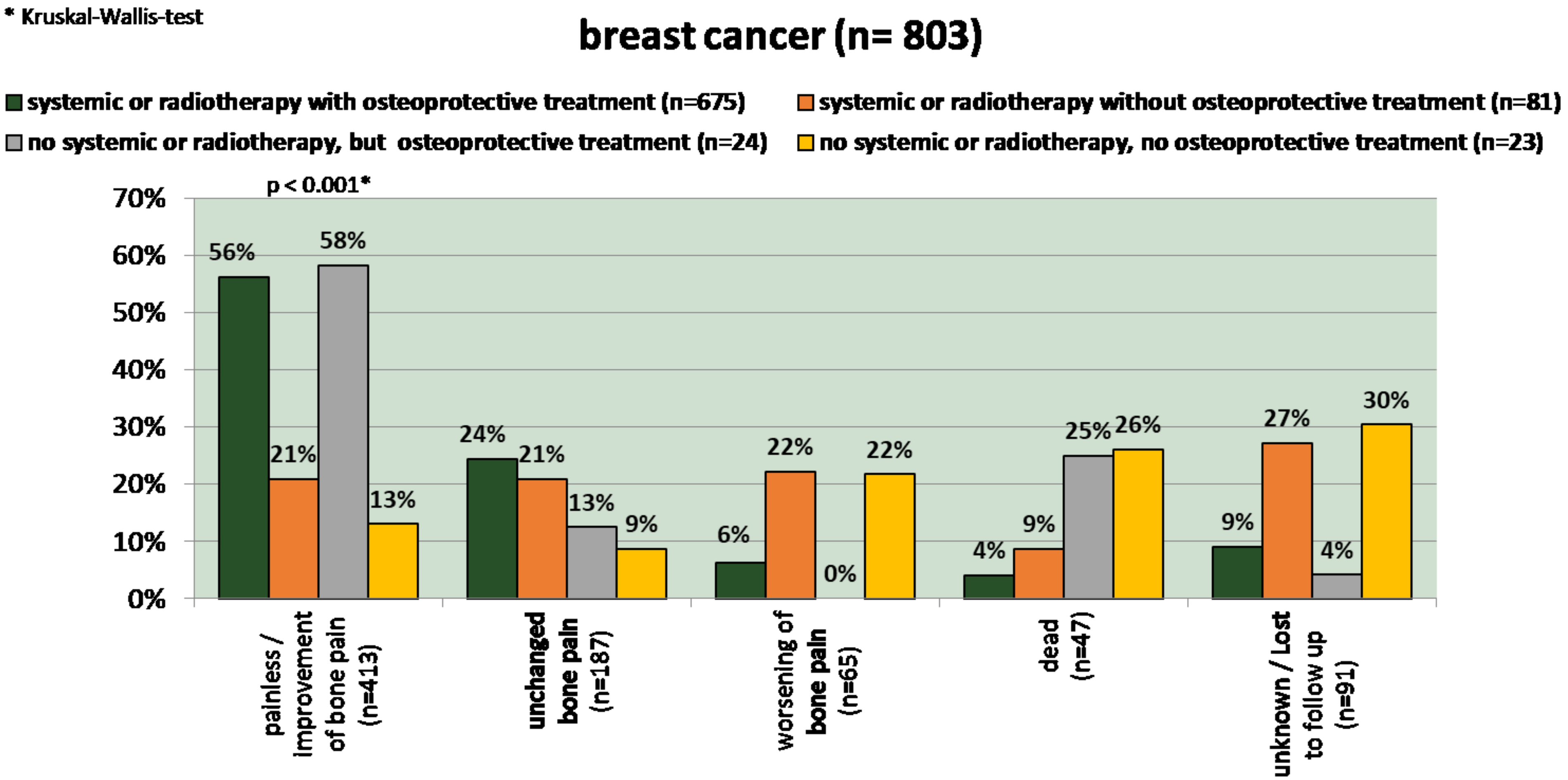

Figure 4D: systemic or radiotherapy, osteoprotective treatment and bone pain

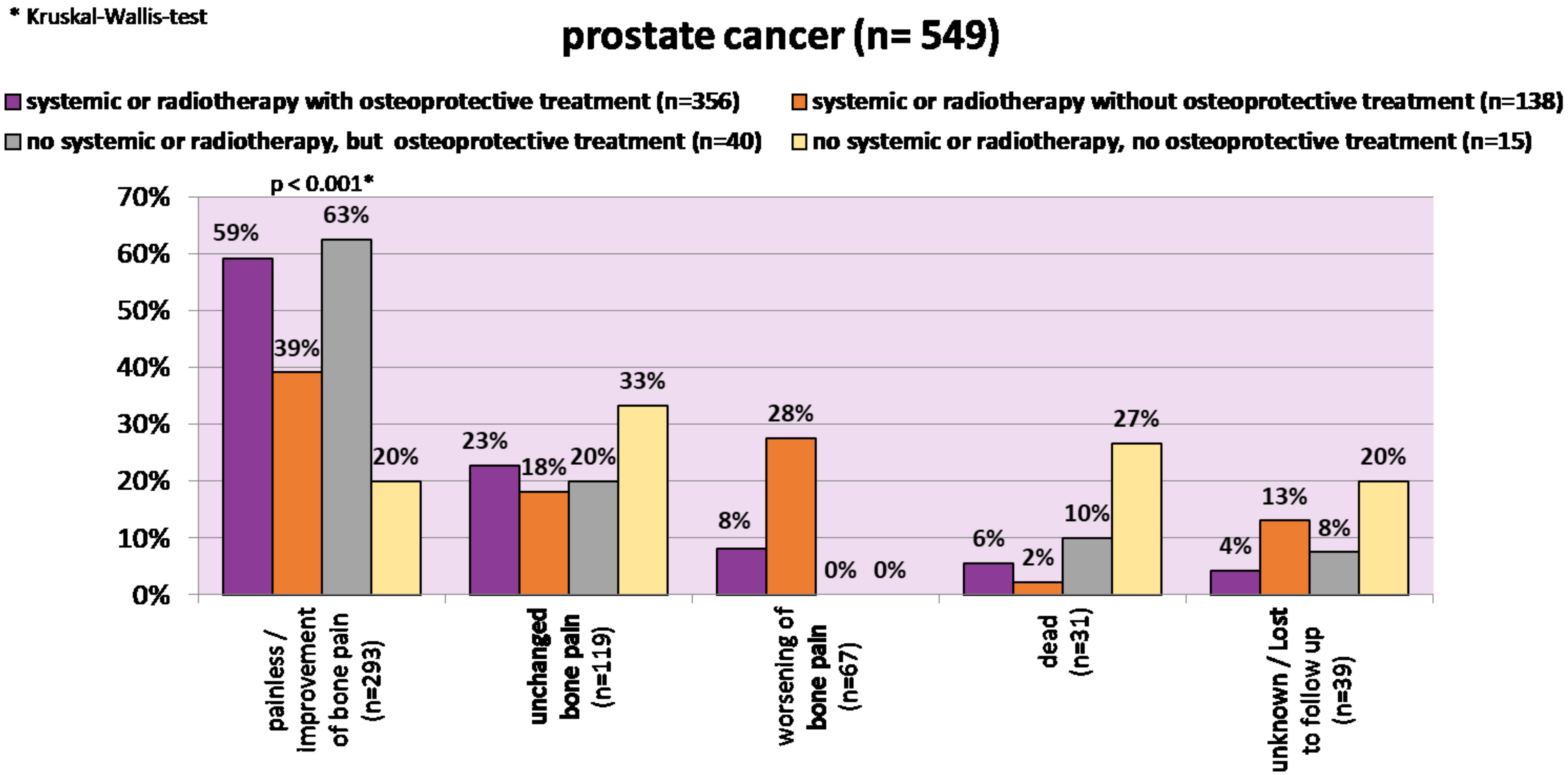

Supplement: Supplementary file 3 — (PDF 345 kb) [file 520_2019_5018_MOESM3_ESM.pdf]
